# Embryonic Cell Grafts in a Culture Model of Spinal Cord Lesion: Neuronal Relay Formation Is Essential for Functional Regeneration

**DOI:** 10.3389/fncel.2016.00220

**Published:** 2016-09-21

**Authors:** Anne Tscherter, Martina Heidemann, Sonja Kleinlogel, Jürg Streit

**Affiliations:** Department of Physiology, University of BernBern, Switzerland

**Keywords:** multielectrode arrays, patch clamp, optogenetics, organotypic culture, halorhodopsin

## Abstract

Presently there exists no cure for spinal cord injury (SCI). However, transplantation of embryonic tissue into spinal cord (SC) lesions resulted in axon outgrowth across the lesion site and some functional recovery, fostering hope for future stem cell therapies. Although *in vivo* evidence for functional recovery is given, the exact cellular mechanism of the graft support remains elusive: either the grafted cells provide a permissive environment for the host tissue to regenerate itself or the grafts actually integrate functionally into the host neuronal network reconnecting the separated SC circuits. We tested the two hypotheses in an *in vitro* SC lesion model that is based on propagation of activity between two rat organotypic SC slices in culture. Transplantation of dissociated cells from E14 rat SC or forebrain (FB) re-established the relay of activity over the lesion site and thus, provoked functional regeneration. Combining patch-clamp recordings from transplanted cells with network activity measurements from the host tissue on multi-electrode arrays (MEAs) we here show that neurons differentiate from the grafted cells and integrate into the host circuits. Optogenetic silencing of neurons developed from transplanted embryonic mouse FB cells provides clear evidence that they replace the lost neuronal connections to relay and synchronize activity between the separated SC circuits. In contrast, transplantation of neurospheres (NS) induced neither the differentiation of mature neurons from the grafts nor an improvement of functional regeneration. Together these findings suggest, that the formation of neuronal relays from grafted embryonic cells is essential to re-connect segregated SC circuits.

## Introduction

Transplantation of embryonic cell grafts has been actively investigated for the treatment of spinal cord injury (SCI) over several decades (Mothe and Tator, 2013). The partial success of this approach is based on two possible regenerative mechanisms mediated by the grafted cells. Either grafted embryonic cells provide a permissive environment to support the regeneration and function of the host tissue itself (Cao et al., 2005) or the grafts may differentiate into neurons that functionally reconnect the separated parts (Bonner and Steward, 2015). Favoring the first option is the observation that propriospinal axons are able to form novel circuits after incomplete SCI (Bareyre et al., 2004; Courtine et al., 2008). On the other hand, embryonic grafts originating from central nervous tissue were shown to provide growth of host axons and the formation of synapses with the grafted cells (Mitsui et al., 2005; Medalha et al., 2014; Kadoya et al., 2016) in the injured spinal cord (SC), favoring the second option. Similarly, cell suspension grafts from E14 SC were shown to survive in the presence of growth factors and differentiate into neurons that grew long-distance axons forming synapses with the host tissue (Lu et al., 2012). Abematsu et al. (2010) even demonstrated in an adult SCI mouse model some functional regeneration of hind limb locomotion after E14 cell engraftment and induction of graft cell differentiation into neurons by addition of valproic acid. Nonetheless, a direct proof that this functional regeneration is based on newly formed neuronal relays by the grafted cells is still lacking.

Fetal nervous tissue contains a mixed population of neural stem cells, neural restricted precursors and glial restricted precursors, which may differentiate into astrocytes, oligodendrocytes or neurons (Lepore and Fischer, 2005). As the number of cells recovered from fetal tissue dissociation is generally low, the number of isolated neural stem cells is often augmented *in vitro* by growing the harvested cells in the presence of growth factors like epidermal growth factor (EGF) and fibroblast growth factor (FGF) into neurospheres (NS), free-floating cell aggregates, before implantation. However, cells from NS that were grafted into the injured SC more likely differentiate into glial cells than into neurons (Enzmann et al., 2006) with a slightly higher neuron yield for forebrain (FB)-derived NS compared to SC-derived NS (Watanabe et al., 2004).

We have recently developed an *in vitro* model that allows the investigation of functional regeneration after SC lesions. It consists of two SC slices cultured together on a multi-electrode array (MEA). After a few days *in vitro* (DIV), the two slices form synaptic connections that relay spontaneous electrical activity from one slice to the other, synchronizing the activity between the slices (Heidemann et al., 2014). At DIV21, the two slices are mechanically separated, which terminally abrogates their synchronization as the regenerative capability is lost at this age *in vitro*.

Here, we inserted embryonic cell suspensions or NS into the lesion site at DIV21 to test for their potential to functionally reconnect the two slices and thus synchronize their activity. The *in vitro* model system allowed us to investigate in detail the activity propagation between the two host SC slices and the grafted cells. By temporally probing the activity spread throughout the model system and optogenetically silencing the grafted cells we were able to pinpoint functional regeneration clearly to the relay action of transplanted cells that had differentiated into mature neurons.

## Materials and Methods

### Animals and Tissue Isolation

SCs and FBs were obtained from 14-day-old rat embryos (E14) and newborn rats (P1) from either Wistar rats (Janvier, Le Genest St. Isle, France) or Lewis rats expressing green fluorescent protein (GFP) ubiquitously in most organs (GFP rats, kindly obtained from Professor S. Leib, University of Bern; Inoue et al., 2005). For optogenetic experiments, heterozygous Nes-cre (B6.Cg-Tg(Nes-cre)1Kln/J, Jackson Laboratories, Bar Harbor, ME, USA) mice were crossed with homozygous eNpHR (Ai39, B6;129SGt(ROSA)26Sortm39(CAG-hop-/EYFP)Hze/J, Jackson Laboratories, Bar Harbor, ME, USA) mice to yield Nes-eNpHR embryonic mice expressing the yellow light activated chloride pump eNpHR3.0 fused to EYFP in all neurons. The embryos were delivered by cesarian section from deeply anesthetized mothers (300 mg/kg KG pentobarbital i.p., Streuli Pharma SA, Switzerland). Embryos and newborns were sacrificed by decapitation and the mother animals by the injection of pentobarbital. Animal care was in accordance with guidelines approved by Swiss local authorities (Amt für Landwirtschaft und Natur des Kantons Bern, Veterinärdienst, Sekretariat Tierversuche, approval Nr. 52/11 and 35/14). These guidelines are in agreement with the European Community Directive 86/609/EEC.

### Preparation of Organotypic Spinal Cord Cultures

**Figure 1:**
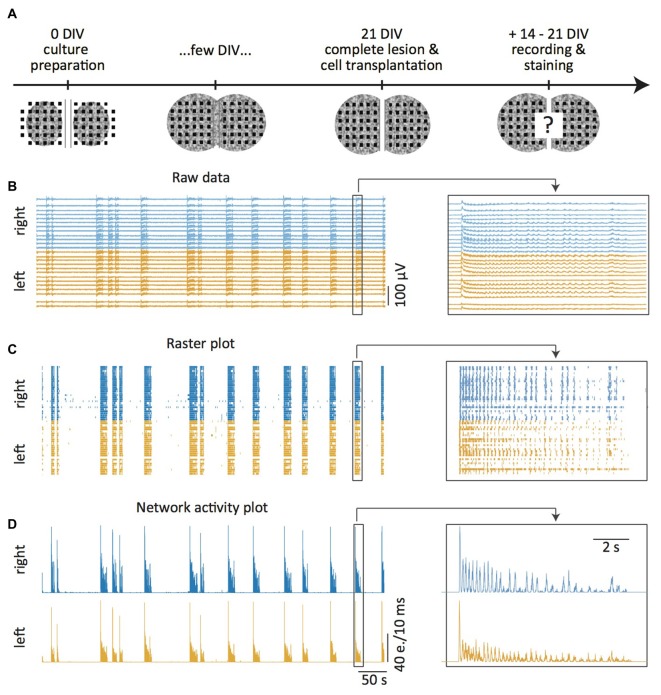
**Experimental setup. (A)** *In vitro* model: two spinal cord (SC) slices are placed on both sides of a 300 μm wide central groove on a multi-electrode array (MEA) at days *in vitro* 0 (DIV0). Within a few days, newly outgrowing axons connect the two slices. At DIV21 the newly generated connections are transected with a scalpel and embryonic cells grafted into the lesion site. Two to three weeks later the slice activity is measured, the tissue fixed and immunohistochemically stained. **(B–D)** MEA recordings 14–21 DIV after slice transection. Raw data under disinhibition (**B**, 10 μM gabazine and 1 μM strychnine) and offline detected neuronal activity displayed as time markers (“events”) in a raster plot **(C)**. Network activity plots representing total activity (**D**, e. = events). The magnification on the right shows a burst pair. The “left” and “right” refer to the left and right slices on the MEA.

**Figure 2:**
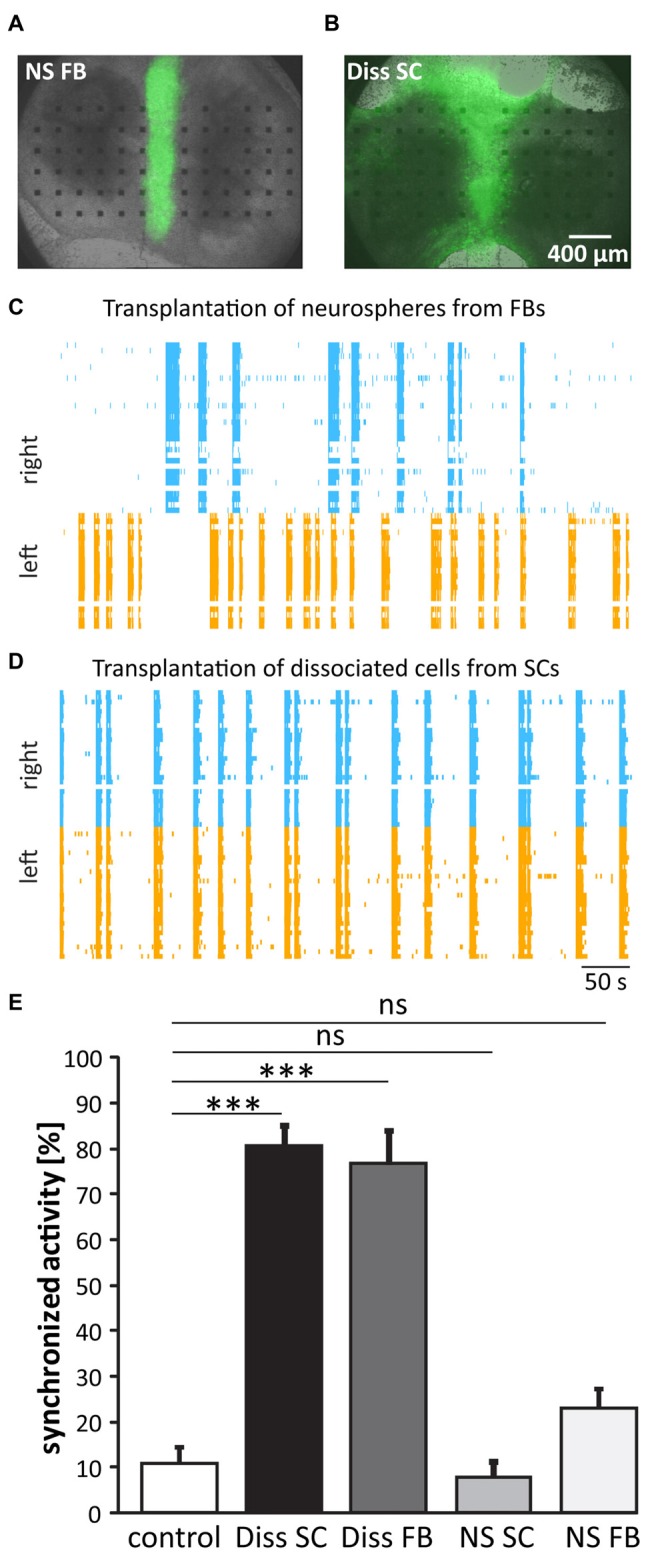
**Functional regeneration after cell transplantation. (A,B)** Pictures of SC cultures 3 weeks after the grafting of forebrain (FB) neurospheres (NS) and dissociated spinal cord (Diss SC) from green fluorescent protein (GFP) rat embryos. **(C,D)** Representative raster plots of cultures that received FB NS **(C)** or Diss SC cells **(D)**. Only in the latter case **(D)** activity between the slices was re-synchronized. **(E)** Quantification of burst synchronization with cell grafts from different sources. Transplantation of dissociated cell suspension originating from both, SCs and FBs, significantly increased burst synchronization over the control group. ns = not significant; Control (lesion in absence of cell transplantation), *n* = 46; Diss SC, *n* = 19; Diss FB, *n* = 11; NS SC, *n* = 10; NS FB, *n* = 24; ***p < 0.001.

Organotypic SC cultures were prepared by isolating the backs of Wistar rat embryos from their limbs and viscera and cutting the backs into 225–250 μm thick transverse slices with a tissue chopper (Heidemann et al., 2015). After dissecting the SC slices from the surrounding tissue two of them were fixed next to each other on top of a MEA (Qwane Biosystems, Lausanne, Switzerland) by using reconstituted chicken plasma coagulated by thrombin (both Sigma-Aldrich, Switzerland). The MEAs had a custom-made design with two areas of 34 electrodes separated by a 300 μm wide central groove without electrodes and insulation layer (see Figures 1A, 2A). The groove allowed mechanical interventions without destroying the lead and insulation layers of the MEA. The slices were arranged with the ventral parts facing each other on either side of the groove at a distance of roughly 300 μm. The cultures were maintained in sterile plastic tubes containing 3 ml of nutrient medium [79% DMEM with Glutamax (Life Technologies, Switzerland), 10% horse serum (Gibco, BRL, Life Technologies AG, Switzerland), 10% H_2_O and 5 ng/ml 2.5S nerve growth factor (Sigma-Aldrich, Switzerland)] and incubated in roller drums rotating at 1 rpm in a 5% CO_2_-containing atmosphere at 36.5°C (Tscherter et al., 2001). Half of the medium was replaced once or twice per week.

### Preparation of E14 Forebrain and Spinal Cord Dissociated Cells for Implantation

Single cell suspensions were prepared under sterile conditions from either the SCs or the FBs of E14 rat embryos from either Wistar rats or GFP-expressing Lewis rats. The tissue of choice (either SC slices without dorsal root ganglions or FBs) was exposed to 0.25% Trypsin-EDTA (Sigma-Aldrich, Switzerland) solution for 20 min at 36.5°C. The Trypsin-EDTA solution was removed and digestion of the tissue was blocked by adding Neurobasal^TM^-medium (Gibco, BRL, Life Technologies AG, Switzerland) containing 10% of horse serum. The tissue was mechanically dissociated by trituration using a pipette tip. Cell suspensions were subsequently filtered to remove tissue clumps. Approximately 15,000 cells were then immediately inserted into the lesion site of 21 DIV old organotypic cultures.

### Neurosphere Preparation

NS were derived from SCs or from FBs of either Wistar rats or of GFP Lewis rats at E14, according to the protocol described in Hofer et al. (2012). Briefly, the dissection was performed in DMEM with 1 mg/ml glucose (Gibco BRL, Life Technologies AG, Switzerland). The tissue was cut into small pieces (around 0.1 mm^3^) and transferred into a solution of defined, serum-free Neurobasal^TM^-medium supplemented with B27, 10 ng/ml bFGF, 10 ng/ml EGF (both PeproTech, Rocky Hill, NJ, USA), 10 μg/ml penicillin-streptomycin (Sigma-Aldrich, Switzerland) and 0.2% heparin (Stem Cell Technologies, Grenoble, France). The cell suspension grew into NS in anti-adhesive poly-HEMA coated petri dishes (Sigma-Aldrich, Switzerland) at 36.5°C in 5% CO_2_ with the medium exchanged every 2–3 days. The NS were passaged every 4–6 days within 2 weeks before they were inserted into the lesion site of 21 DIV old organotypic cultures.

### Lesion and Cell Transplantation

During the first few DIV the organotypic slices grew and fused with each other. We performed a complete lesion with a scalpel to separate them within the groove (Figure 1A). Immediately following the cut, we grafted the cells of choice into the lesion site. In the cases of the dissociated cell suspensions, we inserted 0.5–4 μl (around 15,000 cells). For the NS, we placed 4–6 NS into the lesion site. After insertion, the MEAs were put back very carefully into their empty plastic tubes and transported into the cell culture incubator. Utmost care was taken to prevent agitation as much as possible to allow for the dissociated cells to settle. The cultures were left in the incubator for at least 15 min until half of the medium (DMEM as described above) was added. Cultures were kept horizontally for another 30 min before supplying the full amount of medium and putting them back into the roller drum. The cultures were further incubated for 2–3 weeks until the activity was measured and fixed for immunohistochemical staining.

### MEA Recordings

For the recordings, the MEAs were mounted on an inverted microscope and kept in a bath of extracellular solution containing (in mM) NaCl 145; KCl 4; MgCl_2_ 1; CaCl_2_ 2; HEPES 5; Na-pyruvate 2; glucose 5 (pH 7.4). The recordings were digitized with a PCI6071e A/D-card, visualized and stored on hard disk with custom-made virtual instruments within LabVIEW (both National Instruments, Ennetbaden, Switzerland), as described previously (Tscherter et al., 2001). For “disinhibition”, gabazine (10 μM) and strychnine (1 μM, both Sigma-Aldrich, Switzerland) were added to the extracellular solution. The bath was exchanged after every recording session, which lasted 10 min and was made at room temperature.

MEA recordings were made from organotypic and dissociated cultures (see “Optogenetics” Section). Spontaneous activity in organotypic cultures is organized in bursts. In cultures displaying functional regeneration after lesion and cell transplantation, these bursts propagate from one slice to the other (Figures 1B–D). Bursts can also be induced by electrical stimulation of the culture using an electrode of the MEA. Electrical stimulations were performed with monopolar biphasic stimuli (0.1–0.5 ms, 1–3 V) that were delivered from a custom-made stimulator at 0.1–0.05 Hz. Spontaneous and stimulated activity were recorded in the same way from dissociated cultures.

### Whole-Cell Recordings

Intracellular voltage measurements were obtained from individual neurons originating from GFP rats or Nes-eNpHR mice 2–3 weeks after their insertion into organotypic cultures and from dissociated Nes-eNpHR cells directly grown on MEAs (see “Optogenetics” Section). Patch-clamp recordings were amplified with an Axoclamp 2B amplifier (Molecular Devices Inc., Sunnyvale, CA, USA) and both, visualized and stored using pClamp software (Molecular Devices Inc., Sunnyvale, CA, USA). At the same time, they were also acquired with the same A/D card as the MEA recordings to assure temporal synchronized registration of both data types. The patch pipettes were filled with a solution containing (in mM): K-gluconate (120); KCl (10); EGTA (10); HEPES (10); Mg-ATP (4); Na_2_-GTP (0.3); Na_2_-phosphocreatine (10); pH 7.3 (with KOH). The electrodes had resistances of 3–5 MΩ. No series resistance compensation was applied. Native resting membrane potentials were in the range of −45 mV to −70 mV. Cells with a depolarized potential above −45 mV were discarded. Data were evaluated off-line using custom-made programs in IGOR (WaveMetrics).

### Optogenetics

The SCs and FBs of E13–14 mouse embryos expressing the eNpHR-EYFP fusion protein were dissociated and transplanted into lesioned rat spinal organotypic cultures. To screen for eNpHR expression, green fluorescing mouse embryos were selected under a homemade UV lamp equipped with a GFP filter. In addition, dissociated cultures were prepared directly on MEAs (100,000 cells per MEA) and kept in culture for 3 weeks in presence of Neurobasal^TM^-medium complemented with B27 and glutamax (Gibco, BRL, Life Technologies AG, Switzerland).

Experiments were performed under the same conditions as for the cultures originating from rat tissue. To inhibit the activity of eNpHR-expressing cells, a 591 nm LED (PC Amber LUXEON Rebel LED—140 lm @ 350 mA, Luxeon Star LEDs, Randolph, VT, USA) mounted on a heat sink (SinkPAD-II 25 mm round base) was placed 8 mm below the MEA (Pulizzi et al., 2016; Figure 5A). The light was bundled with a reflector (Dialight 13° 11 mm Circular Beam Optic, Luxeon Star LEDs, Randolph, VT, USA) and the LED was turned on and off over a TTL input. The intensity of illumination was manually controlled over a homemade potentiometer and is expressed in % of the maximal output of the LED.

The onset of a spontaneous burst was detected online using homemade software in LabVIEW (National Instruments, Switzerland). For this we manually selected one electrode per slice that displayed activity in the initial phase of bursts. Bursting activity was subsequently detected in these electrodes using a threshold over the standard deviation (10 ms time window), triggering the immediate on-switching of the LED. Cultures with low burst synchronization (<50%, *n* = 3, see “Analysis of MEA Activity” Section), unreliable online burst detection (used to trigger the light pulses at burst onset, *n* = 1) or unstable electrical stimulation of the bursts were discarded (*n* = 3).

For bursts induced with electrical stimulation, pClamp (Molecular Devices, Sunnyvale, CA, USA) was used to obtain a precise timing between electrical stimulation and light pulse application. For Figure 6F, we extrapolated the light pulse delay leading to the inhibition of the propagation of 50% of the stimulated bursts from the obtained data summarized in Figure 6E for each experiment.

For both stimulated and spontaneous bursts, the LED was turned on for 1–2 s and further illumination was then suppressed for at least 5 s to avoid an increase of the temperature of the LED and the culture.

### Analysis of MEA Activity

A detailed description of the analysis of the spontaneous activity of the SC cultures and the quantification of functional regeneration can be found in Heidemann et al. (2014). Briefly, offline spike detection was applied to the extracellular recordings of the MEA electrodes (Figure 1B) using custom made software based on a standard deviation threshold device and a subsequent discriminator for single unit and multiunit activity without further spike sorting (Tscherter et al., 2001). The detected events were analyzed in IGOR Pro (WaveMetrics, Tigard, OR, USA). They were plotted as time markers in raster plots that display the events for each individual electrode (Figure 1C). The raster plots were then transformed into network activity plots that show the total activity of all selected electrodes per slice in a time window of 10 ms (Figure 1D). The network activity plots were subsequently used to detect activity bursts defined by simultaneous multiunit activity at many electrodes, preceded and followed by silent periods (Figure 1). These data were used to determine activity synchronization as the percentage of bursts that propagated from one slice in the culture to the other (see Figures 1, 2).

### Immunohistochemistry

Immunohistochemical staining was performed as described in Heidemann et al. (2014). Anti-doublecortin (DCX, 1:1000, Millipore) was used to detect recently formed neurons or migrating and differentiating neuroblasts (Korzhevskii et al., 2009). Anti-β-III-tubulin (1:2000, Promega) labels dividing and postmitotic neurons (Menezes and Luskin, 1994; Memberg and Hall, 1995) and NeuN (1:200, Millipore) stains the nuclei of differentiated mature neurons (Cifra et al., 2012). As secondary antibodies, we applied AlexaFluor488 (1:1000, Invitrogen) and AlexaFluor568 (1:500, Invitrogen).

### Statistics

Results are expressed as mean ± SEM. Values were set as significant where *p* < 0.05 (*), very significant where *p* < 0.01 (**), and extremely significant where *p* < 0.001 (***). Statistics were calculated from raw data with InStat (GraphPad Software Inc, La Jolla, CA, USA). The differences among several groups were assessed with one-way ANOVA/non-parametric, two-tailed Kruskal-Wallis tests.

## Results

### Dissociated Cell Suspensions Mediate Functional Regeneration

We first compared the ability of different cell grafts from E14 GFP rats to promote functional regeneration: (i) dissociated spinal cord (Diss SC); (ii) dissociated forebrain (Diss FB); (iii) NS from SC; and (iv) NS from FB. We transected *in vitro* at DIV21 the functional connections between the two SC slices and grafted the cells of choice into this lesion site. We assessed functional regeneration 2–3 weeks later by quantifying the activity propagation between the two host slices. The combination of the slice culture model with grafted GFP-positive cells enabled us to have direct experimental access to both, the host tissue by electrical MEA recordings and the grafted cells by their GFP fluorescence that enabled us to identify the grafted cells for patch-clamp recordings.

Two to three weeks after transplantation, cells from dissociated tissue had distributed throughout the organotypic cultures, whilst the cells from the NS stayed within the lesion site, with few having migrated marginally (Figures 2A,B). The functional recovery was at the same time point assessed by recording the network activity of the host tissue and calculating the percentage of synchronized bursts between the two slices. To stabilize spontaneous busting, experiments were conducted under disinhibition (10 μM gabazine and 1 μM strychnine, see also Heidemann et al., 2014). Recordings of the network activity revealed a highly synchronized activity pattern in cultures having received dissociated cells from either the SC or the FB (80 ± 4.3%, *n* = 19 and 79 ± 7.9%, *n* = 8; Figures 2C–E; one-way ANOVA/non-parametric, two-tailed Kruskal-Wallis test), but not in those that had received NS (spinal: 7.6 ± 3.3%, *n* = 10 and FB: 23 ± 4.2%, *n* = 24; one-way ANOVA/non-parametric, two-tailed Kruskal-Wallis test). The results clearly favor dissociated cells and not NS as graft sources for functional regeneration.

### Transplanted Dissociated Cells Differentiate into Mature Neurons

Next, we investigated if functional regeneration correlates with graft cell differentiation into neurons. If so, this would be in line with the possibility that the differentiated neurons from the grafted cells functionally connect with the host tissue und thereby relay activity from one slice to the other. Otherwise, it would rather favor the idea that the grafted cells act as supporters for host tissue regeneration, i.e., stimulating the sprouting of host neurons by releasing trophic factors. To distinguish between these possibilities we determined the neuronal identity of the transplanted cells with immunohistochemical stainings 3 weeks after cell grafting.

Anti-DCX was used to identify neuroblasts, anti-β-III-tubulin to identify newly generated immature postmitotic neurons and anti-NeuN to identify mature neurons (von Bohlen Und Halbach, 2007).

**Figure 3:**
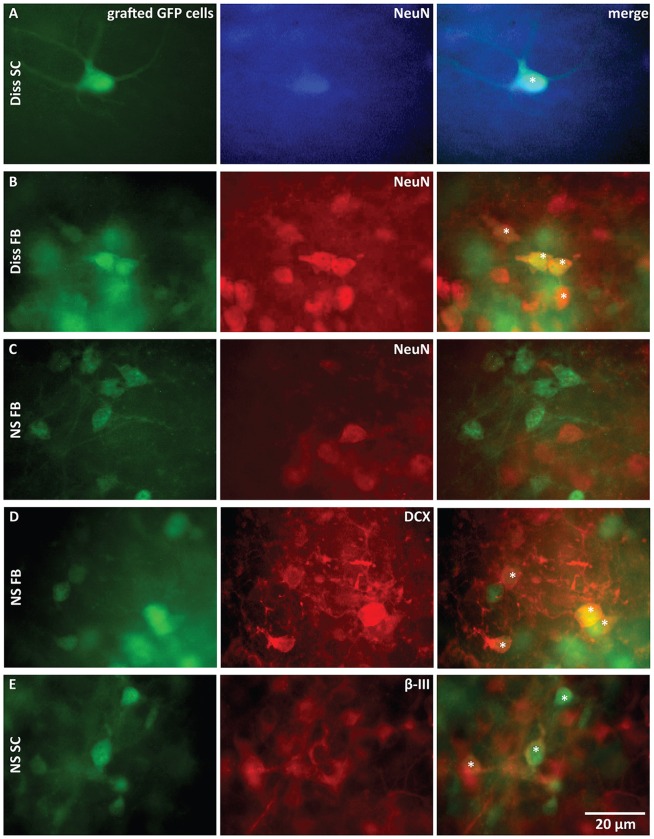
**Immunohistochemical characterization of grafted cells 3 weeks after transplantation. (A–E)** Comparison of GFP stainings of transplanted cells with stainings for mature neurons **(A–C)**, neuroblasts **(D)** and postmitotic neurons **(E)**. Stars (*) mark double-stained cells. Only dissociated cells (Diss) from FB and SC gave rise to mature neurons. Anti-β-III-tubulin (β-III): postmitotic neurons, anti-Doublecortin (*DCX*): neuroblasts, anti-NeuN: mature neurons, NS, neurospheres.

**Figure 4:**
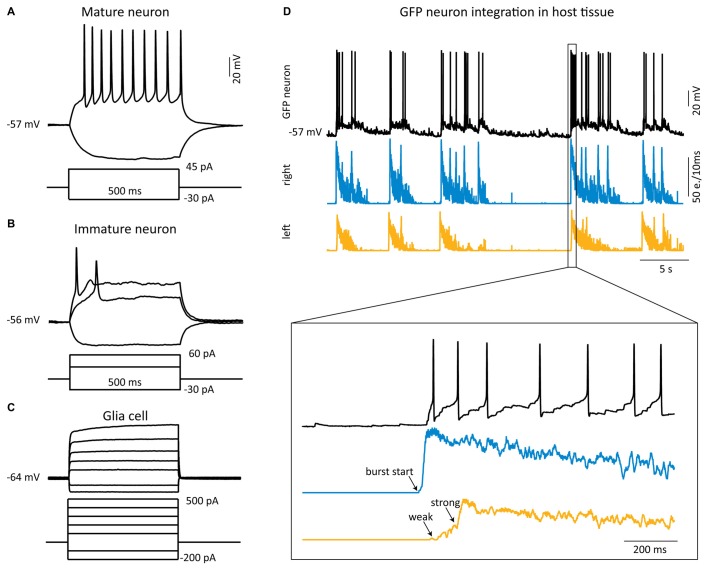
**Functional integration of grafted matured neurons into the host tissue. (A–C)** Whole-cell current-clamp recordings (black traces) from single GFP cells from different sources 2–3 weeks after transplantation. **(A)** Example of sustained action potential firing induced by a depolarizing current step in a mature neuron (dissociated cell from SC). **(B,C)** Typical electrophysiological behavior of an immature neuron **(B)** and a glial cell **(C)**, both from FB NS. **(D)** Example of a simultaneous whole-cell recording (upper black trace) from a grafted Diss SC neuron with MEA recordings from the host tissue slices. The burst firing starts in the right slice (middle trace) and propagates to the left slice (lower trace), which displays first some weak, then strong bursting activity. The GFP neuron receives strong synaptic inputs from the right, leading slice resulting in action potential firing. e. = events.

NeuN-labeled grafted GFP cells were only found in the two dissociated cell populations from SC (Figure 3A) and from FB (Figure 3B). Grafted cells derived from NS were negative for anti-NeuN (SC: not shown; FB: Figure 3C), but stained positive for anti-DCX (Figure 3D) or anti-β-III-tubulin (Figure 3E), indicating that these neurons remained immature. This difference in neuronal maturation between dissociated cell grafts and NS grafts was functionally confirmed by whole-cell recordings from grafted cells. Fast and repetitive action potential firing in response to current injection, indicative of a mature neuron, was only found in dissociated graft cells (9 of 18 recorded cells; Figure 4A). Two dissociated grafted cells responded with single small and slow action potentials in response to prolonged depolarizing current injection and were therefore considered as immature neurons. The remaining seven cells displayed typical glial electrophysiological behavior. In line with above morphological results, no grafted cells from NS displayed mature neuronal characteristics (five cultures) and recordings could be clearly allocated to immature neurons (*n* = 3, Figure 4B) or glia cells (*n* = 8, Figure 4C).

### Grafted Matured Neurons can Functionally Integrate into The Host Tissue

To investigate if the grafted matured neurons functionally integrate into the host circuitry, we simultaneously acquired the activity of the host SC cultures by MEA recordings and of the grafted GFP neurons by patch-clamp recordings. All 10 patched GFP neurons responded with EPSPs to the activity of the host tissue and the synaptic drive was in six of them strong enough to generate action potentials. In most of these neurons (8/10) the EPSP activity temporally correlated with the network activity in the slice where the grafted neuron was located, suggesting that they received their input from this slice. The set-up allowed us to study in detail the activity propagation between the host tissue slices and the grafted cells: activity was onset by a burst in one of the SC slices, referred to as the leading slice, which was followed by activity in the grafted GFP neuron (we focused on grafted neurons located in the leading slice, *n* = 4), firing action potentials with a delay of 111 ± 20 ms. With a longer delay (170 ± 55 ms) relative to the burst activity in the leading slice the opposing slice (referred to as the follower slice) responded with strong bursting activity (see Figure 4D). Interestingly, bursting activity in the follower slice could not be induced by simply forcing one GFP neuron to fire action potentials by depolarizing current injection, suggesting that the drive from one neuron is insufficient to drive the follower slice, as expected.

Our results show that the grafted matured neurons functionally integrate into the host tissue and potentially transfer activity from the leading to the follower slice.

### Optogenetic Silencing of The Cells Used for Transplantation

To investigate the role of the grafted neurons in relaying activity between the two slices in more detail, we sought to inhibit the graft cells time-locked to the bursting activity of the host tissue using the optogenetic silencer halorhodopsin (eNpHR; Zhang et al., 2007). To acquire a homogeneous embryonic graft cell population that expressed the yellow-light driven chloride pump, we crossed two available transgenic mouse lines (Nes-cre and eNpHR mice) to produce offsprings that expressed the eNpHR-EYFP fusion protein ubiquitously in precursor neurons from FB and SC (Nes-eNpHR mice: see “Materials and Methods” Section). EYFP expression allowed for visualization of the grafted eNpHR-expressing cells in the host tissue.

**Figure 5:**
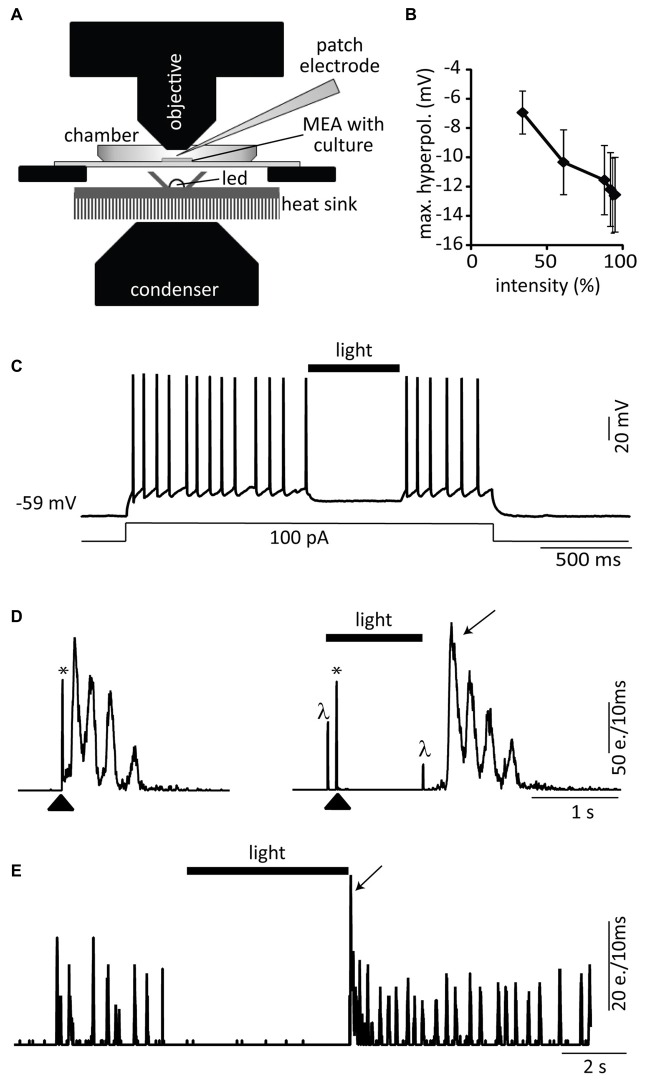
**Optogenetic silencing with eNpHR. (A)** Setup configuration: a 591 nm stimulation LED was placed just below the MEA located on an upright microscope. Dissociated cells originating from FBs of Nes-eNpHR mice (**B–D**, E14 and E, P1) were directly cultured on MEAs in absence of host tissue. **(B)** Yellow light pulses (1–2 s) of increasing intensities (expressed in % of maximum) lead to progressively increased hyperpolarizations (*n* = 5). **(C)** Representative current-clamp trace showing the strong yellow light mediated inhibition of a current-step induced train of action potentials. **(D)** Electrical stimulation through an electrode of the MEA (arrowhead, left graph, network activity plot). One second long yellow light pulses starting 100 ms before the MEA stimulation suppressed bursts (right graph). **(E)** Example of spontaneous network activity inhibited by sustained yellow light illumination. Both, electrical stimulation (*) and onset as well as offset of the light pulses (*λ*) induce electrical artifacts. Activity rebound after light pulse (arrows in **D,E**). e. = events. Black bars (light). The LED intensity was set at 94% of its maximal light output in **(C–E)**.

In a first set of experiments we cultured dissociated cells from E14 Nes-eNpHR mouse FBs directly on MEAs to form networks within 3 weeks and to test whether eNpHR activation with yellow light (591 nm, Figure 5A) reliably suppressed action potential firing and network activity in these cultures. Single cell recordings confirmed that yellow light pulses hyperpolarized Nes-eNpHR neurons in a light-intensity dependent manner (*n* = 5, Figure 5B). Yellow light pulses (94% of LED maximum) substantially hyperpolarized 8 out of 10 eNpHR neurons (11.8 ± 1.7 mV, *n* = 8) and reliably inhibited action potential firing induced by a depolarizing current step (Figure 5C). In the remaining two neurons yellow light also induced hyperpolarizations, however of smaller amplitude (<6 mV). Using MEA recordings we next tested in the same dissociated E14 mouse FB cultures if induced bursting network activity of Nes-eNpHR cells could be silenced as a whole by yellow light. Since spontaneous activity was in most Nes-eNpHR cultures small, compound burst firing of the whole culture was elicited by stimulation with an electrode of the MEA. The yellow light was turned on 100 ms before electrical stimulation and prevented bursts in 6 out of 11 cultures (Figure 5D). Spontaneous bursting network activity was also abolished in 3/3 dissociated cultures from neonatal (P1) Nes-eNpHR mice (Figure 5E). We conclude from above experiments that yellow-light inhibition of dissociated Nes-eNpHR cells reliably abolishes generation of action potential firing.

### Optogenetic Silencing of Grafted Neurons Blocks the Propagation of Activity Between The Host Slices

**Figure 6:**
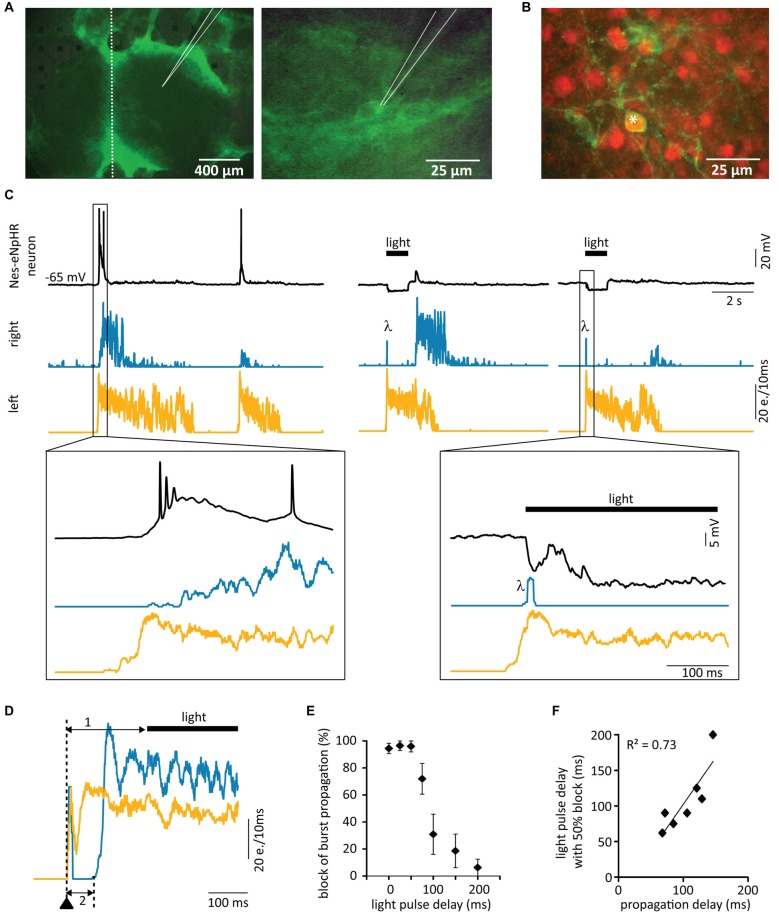
**Grafted matured neurons relay network activity.** Dissociated fluorescing cells originating from FBs of Nes-eNpHR mice (E14) were transplanted into lesioned rat SC cultures at DIV21. **(A)** Photomicrographs 3 weeks after the lesion showing the whole culture (left) and a transplanted Nes-eNpHR neuron (right). Location of patch electrode indicated, dotted line represents site of lesion. **(B)** Labeling against NeuN (red) of Nes-eNpHR-EYFP cells (green) confirms their maturity. Star (*) marks double-stained cell. **(C)** Simultaneous MEA recordings from the SC slices and single-cell patch-clamp recordings from Nes-eNpHR grafted neurons. Left: network activity in the left slice precedes bursting activity in the grafted neuron, which in turn induces activity in the right slice. Right: this strictly unidirectional activity propagation through the grafted neuron acting as a relay between the slices is verified by optogenetic activity suppression in the grafted neuron. Bursting activity in the left slice triggered the switching on of yellow light, preventing activity in the grafted neuron resulting in a lack of activity in the right slice. *λ* artifacts due to the light onset. **(D)** Activity induction in the left slice (arrowhead) with an electrode of the MEA. The yellow light pulse was applied 150 ms after stimulation (1), which did not prevent activity relay into the follower slice (2). Note that the light has no influence on the bursting activity of the two slices confirming that this activity originates from the host tissue. **(E)** Quantification of the influence of the light-delay on the amount of block of activity propagation between slices (*n* = 7). **(F)** Mean propagation delay vs. pulse delay leading to 50% propagation showing that the maximum light pulse delays inducing propagation block are directly related to the activity propagation delay between the slices. e. = events. LED 94% of maximum.

In the final step, we combined our organotypic *in vitro* model with optogenetic silencing of grafted neurons and transplanted dissociated E14 Nes-eNpHR FB cells into the lesion site of DIV21 rat organotypic SC cultures. MEA network activity recordings of the rat host tissue revealed 67.8 ± 7.9% of burst synchronization between the two slices 2**–**3 weeks after transplantation (*n* = 14) and immunohistochemical staining against NeuN confirmed the presence of mature Nes-eNpHR neurons (Figures 6B,C). Single cell recordings from Nes-eNpHR neurons revealed that they had integrated into the host circuits as they showed synchronized activity with the host networks (Figures 6A,C).

Since the transplantation of Nes-eNpHR cells recovered synchronized activity between the slices and optogenetics enabled us to inhibit action potential firing in the grafted cells by yellow light, we used this setup to test whether the grafted matured neurons were indeed able to relay neuronal activity between slices. In fact, if this was the case, yellow light pulses should prevent the propagation of spontaneous or stimulated bursts thus interrupting synchronization of activity.

In all experiments 1–2 s long yellow light pulses were applied at the onsets of bursts. In seven out of nine cultures illumination effectively blocked the transmission of spontaneous bursts from one slice to the other, with ≥85% of bursts not reaching the other slice. Simultaneous single cell recordings confirmed that yellow light pulses induced a hyperpolarization in grafted Nes-eNpHR cells preventing action potential firing in response to bursting activity of the leading slice (Figure 6C). In control experiments with lesioned cultures that had received FB cells from GFP rats or from non-transgenic mouse littermates not expressing eNpHR, illumination never blocked the propagation of bursts (data not shown).

Propagation of activity from the leading slice to the follower slice occurred with variable delays up to 170 ms. To investigate how the timing of the light pulse is related to the propagation delay, we elicited bursts by electrical stimulation of one slice and applied light pulses with variable delays with respect to the stimulation (Figure 6D). In 9 of 11 cultures, light pulses concomitantly with stimulation reliably blocked the propagation of ≥85% of the bursts to the opposing slice. Delayed light application also blocked the burst propagation between the slices (*n* = 7), however, only as long as the light pulse occurred before activity induction in the follower slice (Figures 6E,F). This shows that the silencing of the grafted neurons only suppressed the propagation of bursts between the slices but not the maintenance of activity during the bursts (see Figure 6D). These results therefore clearly confirm that the grafted neurons present the fundamental building blocks to relay bursting activity between two SC slices.

## Discussion

The transplantation of embryonic cell grafts has proven to be a promising approach for the treatment of SCI. For example, transplantation of fetal neural cells into the lesioned SC of an adult mouse clearly led to functional recovery of hind limb locomotion after the grafted cells had differentiated into mature neurons (Abematsu et al., 2010). However, the cellular mechanisms underlying such physical recovery remained elusive; did the grafted cells simply release trophic factors stimulating the regeneration of host circuits or do the neurons that differentiate from the grafted cells actually embody the newly formed functional connections? Latter presents a much more specific role for the cellular grafts. Here we used the functional re-connection between two segregated SC explants as an *in vitro* model to address this question. Our results suggest that the formation of neuronal relays from the grafted cells is essential for functional re-connection.

### Functional Re-Connection of Spinal Cord Circuits by Grafted Embryonic Cells *In Vitro*

Most studies investigating the effects of embryonic grafts to improve regeneration after SCI *in vivo* use a great variety of SCI rodent models ranging from complete SC transections to hemisections, incomplete lesions and contusions (Silva et al., 2014). Since the degree of spontaneous functional recovery highly depends on the type of lesion, the outcomes are often difficult to compare between the studies. We therefore recently designed an *in vitro* SCI model to study regeneration in a well-defined environment (Heidemann et al., 2014). In our model, two adjacent SC slices from E14 rat are co-cultured and readily form functional units with propagating activity between the two slices, leading to synchronized activity as found in isolated SC networks from neonatal rats under similar conditions (Bracci et al., 1996). After disconnecting the two units by complete transection at DIV21 neural connections can no longer be re-established to synchronize activity between the slices, thus resembling the adult situation *in vivo*. Our previous findings suggest that this impairment is not mainly based on axonal growth limitations since axons from one slice were able to grow into the other slice. Furthermore, important inhibitors of axonal regeneration like NogoA or glial scars are not functional in the model (Heidemann et al., 2014). The limitations in functional recovery are therefore more likely based on limitations in the formation of functional connectivity including synapse formation. Although our *in vitro* model lacks several elements of SCI *in vivo* as glia scar formation, vascularization of the tissue or an immune response, it is well suited as a screening system to test the potential of various neural transplants in their capability to form electrophysiologically functional relays.

Here, we re-established functional connectivity between the transected slices by transplanting dissociated E14 SC or FB cells into the lesion site at DIV21. We were able to demonstrate that transplanted cells differentiate into mature spiking neurons that functionally integrate into the host circuits and successfully re-synchronize neural activity between the slices. It is remarkable that the origin of the graft cells—SC *vs*. FB and rat *vs*. mouse—did not influence the outcome. Progenitors from both origins, SC and FB, have been shown to improve regeneration from SCI *in vivo* (Watanabe et al., 2004; Abematsu et al., 2010; Lu et al., 2012).

Optogenetic silencing of the differentiated grafted cells further enabled us to clearly prove their role as neuronal activity relays that mediate functional recovery. We propose that this mechanism is relevant for the *in vivo* situation as it has been shown that the recovery of motor functions in adult mice is highly improved under conditions that favor the differentiation of mature neurons from the graft (Abematsu et al., 2010).

As recovery of functional coupling between the two transected slices reliably occurred under conditions when GABAergic and glycinergic inhibition was blocked (disinhibition), we suggest that the relay was mediated by glutamatergic excitatory connections. In line with this hypothesis, we have previously shown in the same model that cholinergic connections are not involved in re-coupling of slices (Heidemann et al., 2015). Thus at least some of the graft precursors differentiate into glutamatergic neurons. These new neurons probably resemble typical newborn neurons in the SC or hippocampus with strongly depolarizing GABAergic inputs (Ben-Ari, 2001). Similar to newborn granule cells in the dentate gyrus, they may be privileged in activation by combined GABAergic and glutamatergic inputs (Heigele et al., 2016) and therefore may have a high impact on re-coupling between the slices. Our finding that bursts reliably propagate between the slices when GABA/glycine receptors are blocked does not exclude a contribution of GABA and/or glycinergic connections during the formation of new circuits between the spinal slices and the transplanted cells.

### Neuronal Relay Formation From Grafted Embryonic Cells May Underlie Functional Recovery *In Vivo*

Grafting of embryonic SC tissue into the injured SC has been used for several decades as a potential strategy to improve functional regeneration (Reier et al., 1983, 1986). Such grafts were hypothesized to improve the growth and regeneration of host axons and circuits (Bregman and Reier, 1986) and/or to provide intraspinal relay circuits (for review see Bonner and Steward, 2015). Evidence for the latter hypothesis requires differentiation of graft cells into mature neurons that innervate the host tissue as well as the formation of functional synapses between host axons and these graft neurons (Kadoya et al., 2016). Several studies have shown by immunohistochemical labeling that mature neurons can be acquired from embryonic cells derived from: (i) explants (Jakeman and Reier, 1991); (ii) dissociated cells (Lu et al., 2012; Medalha et al., 2014); and (iii) lineage-restricted neural precursors (Lepore and Fischer, 2005). However, differentiation of the grafted cells into mature neurons remained in all studies a challenge. In mixed cultures of a young (DIV7) with an old (DIV21) SC slice we have previously shown that functional connections can form in both directions, suggesting that embryonic grafts can be well integrated into more differentiated circuits (Heidemann et al., 2014).

Improvement of functional regeneration occurred particularly in neonatal animals (Howland et al., 1995; Miya et al., 1997; Diener and Bregman, 1998) and was less reliably observed in adult animals (Bregman and Reier, 1986; Stokes and Reier, 1992; Bonner et al., 2011; Lu et al., 2012; Sharp et al., 2014; Kadoya et al., 2016). Abematsu et al. (2010) reported a greatly enhanced restoration of hind limb function in adult SCI mice after transplantation of neural stem cells that were influenced towards neuronal differentiation by administration of valproic acid. This study emphasizes the importance of graft cell differentiation into mature neurons for functional recovery. In this view, it is not surprising that NS grafts did not improve functional recovery in our model as the graft cells did not differentiate into mature neurons. Also Enzmann et al. (2006) showed previously that graft cells originating from NS mostly differentiate into glial cells, especially in non-neurogenic regions like the SC (Cao et al., 2002; Han et al., 2004). When grafted into a neurogenic region like the hippocampus, we have previously shown that cells from NS are able to differentiate into mature neurons and to functionally integrate into the host circuits (Hofer et al., 2012).

In summary, our results show that in a culture model of SCI, functional regeneration is highly improved by grafting embryonic cells if mature neurons differentiate from the grafts and integrate into the host circuits to relay information across the lesion. This critical role of neuronal differentiation and integration of graft cells for strong functional improvements after a lesion may also hold true for cell transplantation *in vivo*.

## Author Contributions

JS and AT designed the research. SK coordinated the optogenetic part of the study. MH and AT performed research and analyzed the data. All authors wrote, read and approved the final manuscript.

## Funding

This work was supported by the “Novartis Stiftung für medizinisch-biologische Forschung” (n° 15A051 to AT) and the Swiss National Science Foundation (n° 31003A_152807/1 to SK; 31003A_140754/1 to JS).

## Conflict of Interest Statement

The authors declare that the research was conducted in the absence of any commercial or financial relationships that could be construed as a potential conflict of interest.

## References

[B1] Abematsu M. Tsujimura K. Yamano M. Saito M. Kohno K. Kohyama J.. (2010). Neurons derived from transplanted neural stem cells restore disrupted neuronal circuitry in a mouse model of spinal cord injury. J. Clin. Invest. 120, 3255–3266. 10.1172/JCI42957 20714104 PMC2929730

[B2] Bareyre F. M. Kerschensteiner M. Raineteau O. Mettenleiter T. C. Weinmann O. Schwab M. E. (2004). The injured spinal cord spontaneously forms a new intraspinal circuit in adult rats. Nat. Neurosci. 7, 269–277. 10.1038/nn1195 14966523

[B3] Ben-Ari Y. (2001). Developing networks play a similar melody. Trends Neurosci. 24, 353–360. 10.1016/s0166-2236(00)01813-0 11356508

[B4] Bonner J. F. Connors T. M. Silverman W. F. Kowalski D. P. Lemay M. A. Fischer I. (2011). Grafted neural progenitors integrate and restore synaptic connectivity across the injured spinal cord. J. Neurosci. 31, 4675–4686. 10.1523/JNEUROSCI.4130-10.2011 21430166 PMC3148661

[B5] Bonner J. F. Steward O. (2015). Repair of spinal cord injury with neuronal relays: from fetal grafts to neural stem cells. Brain Res. 1619, 115–123. 10.1016/j.brainres.2015.01.006 25591483 PMC4499497

[B6] Bracci E. Ballerini L. Nistri A. (1996). Localization of rhythmogenic networks responsible for spontaneous bursts induced by strychnine and bicuculline in the rat isolated spinal cord. J. Neurosci. 16, 7063–7076. 8824342 10.1523/JNEUROSCI.16-21-07063.1996 PMC6579249

[B7] Bregman B. S. Reier P. J. (1986). Neural tissue-transplants rescue axotomized rubrospinal cells from retrograde death. J. Comp. Neurol. 244, 86–95. 10.1002/cne.902440107 3950092

[B8] Cao Q. L. Howard R. M. Dennison J. B. Whittemore S. R. (2002). Differentiation of engrafted neuronal-restricted precursor cells is inhibited in the traumatically injured spinal cord. Exp. Neurol. 177, 349–359. 10.1006/exnr.2002.7981 12429182

[B9] Cao Q. L. Xu X. M. Devries W. H. Enzmann G. U. Ping P. P. Tsoulfas P.. (2005). Functional recovery in traumatic spinal cord injury after transplantation of multineurotrophin-expressing glial-restricted precursor cells. J. Neurosci. 25, 6947–6957. 10.1523/JNEUROSCI.1065-05.2005 16049170 PMC2813488

[B10] Cifra A. Mazzone G. L. Nani F. Nistri A. Mladinic M. (2012). Postnatal developmental profile of neurons and glia in motor nuclei of the brainstem and spinal cord and its comparison with organotypic slice cultures. Dev. Neurobiol. 72, 1140–1160. 10.1002/dneu.20991 22021114

[B11] Courtine G. Song B. Roy R. R. Zhong H. Herrmann J. E. Ao Y.. (2008). Recovery of supraspinal control of stepping via indirect propriospinal relay connections after spinal cord injury. Nat. Med. 14, 69–74. 10.1038/nm1682 18157143 PMC2916740

[B12] Diener P. S. Bregman B. S. (1998). Fetal spinal cord transplants support growth of supraspinal and segmental projections after cervical spinal cord hemisection in the neonatal rat. J. Neurosci. 18, 779–793. 9425019 10.1523/JNEUROSCI.18-02-00779.1998 PMC6792540

[B13] Enzmann G. U. Benton R. L. Talbott J. F. Cao Q. L. Whittemore S. R. (2006). Functional considerations of stem cell transplantation therapy for spinal cord repair. J. Neurotrauma 23, 479–495. 10.1089/neu.2006.23.479 16629631

[B14] Han S. S. W. Liu Y. Tyler-Polsz C. Rao M. S. Fischer I. (2004). Transplantation of glial-restricted precursor cells into the adult spinal cord: survival, glial-specific differentiation and preferential migration in white matter. Glia 45, 1–16. 10.1002/glia.10282 14648541

[B15] Heidemann M. Streit J. Tscherter A. (2014). Functional regeneration of intraspinal connections in a new *in vitro* model. Neuroscience 262, 40–52. 10.1016/j.neuroscience.2013.12.051 24394955

[B16] Heidemann M. Streit J. Tscherter A. (2015). Investigating functional regeneration in organotypic spinal cord co-cultures grown on multi-electrode arrays. J. Vis. Exp. 103:e53121. 10.3791/53121 26436646 PMC4692611

[B17] Heigele S. Sultan S. Toni N. Bischofberger J. (2016). Bidirectional GABAergic control of action potential firing in newborn hippocampal granule cells. Nat. Neurosci. 19, 263–270. 10.1038/nn.4218 26752162

[B18] Hofer S. Magloire V. Streit J. Leib S. L. (2012). Grafted neuronal precursor cells differentiate and integrate in injured hippocampus in experimental pneumococcal meningitis. Stem Cells 30, 1206–1215. 10.1002/stem.1097 22489030

[B19] Howland D. R. Bregman B. S. Tessler A. Goldberger M. E. (1995). Transplants enhance locomotion in neonatal kittens whose spinal cords are transected–a behavioral and anatomical study. Exp. Neurol. 135, 123–145. 10.1006/exnr.1995.1072 7589324

[B190] Inoue H. Ohsawa I. Muramaki T. Kimura A. Hakamata Y. Sato Y. (2005). Development of new inbred transgenic strains of rats with LacZ or GFP. Biophys. Res. Commun. 329, 288–295. 10.1016/j.bbrc.2005.01.132 15721305

[B20] Jakeman L. B. Reier P. J. (1991). Axonal projections between fetal spinal-cord transplants and the adult-rat spinal-cord–a neuroanatomical tracing study of local interactions. J. Comp. Neurol. 307, 311–334. 10.1002/cne.903070211 1713233

[B21] Kadoya K. Lu P. Nguyen K. Lee-Kubli C. Kumamaru H. Yao L.. (2016). Spinal cord reconstitution with homologous neural grafts enables robust corticospinal regeneration. Nat. Med. 22, 479–487. 10.1038/nm.4066 27019328 PMC4860037

[B22] Korzhevskii D. E. Petrova E. S. Kirik O. V. Otellin V. A. (2009). Assessment of neuron differentiation during embryogenesis in rats using immunocytochemical detection of doublecortin. Neurosci. Behav. Physiol. 39, 513–516. 10.1007/s11055-009-9164-0 19517249

[B23] Lepore A. C. Fischer I. (2005). Lineage-restricted neural precursors survive, migrate and differentiate following transplantation into the injured adult spinal cord. Exp. Neurol. 194, 230–242. 10.1016/j.expneurol.2005.02.020 15899260

[B24] Lu P. Wang Y. Graham L. McHale K. Gao M. Wu D.. (2012). Long-distance growth and connectivity of neural stem cells after severe spinal cord injury. Cell 150, 1264–1273. 10.1016/j.cell.2012.08.020 22980985 PMC3445432

[B25] Medalha C. C. Jin Y. Yamagami T. Haas C. Fischer I. (2014). Transplanting neural progenitors into a complete transection model of spinal cord injury. J. Neurosci. Res. 92, 607–618. 10.1002/jnr.23340 24452691

[B26] Memberg S. P. Hall A. K. (1995). Deviding neuron precursors express neuron-specific tubulin. J. Neurobiol. 27, 26–43. 10.1002/neu.480270104 7643073

[B27] Menezes J. R. L. Luskin M. B. (1994). Expression of neuron-specific tubulin defines a novel population in the proliferative layers of the developing telencephalon. J. Neurosci. 14, 5399–5416. 8083744 10.1523/JNEUROSCI.14-09-05399.1994 PMC6577108

[B28] Mitsui T. Shumsky J. S. Lepore A. C. Murray M. Fischer I. (2005). Transplantation of neuronal and glial restricted precursors into contused spinal cord improves bladder and motor functions, decreases thermal hypersensitivity and modifies intraspinal circuitry. J. Neurosci. 25, 9624–9636. 10.1523/JNEUROSCI.2175-05.2005 16237167 PMC6725721

[B29] Miya D. Giszter S. Mori F. Adipudi V. Tessler A. Murray M. (1997). Fetal transplants alter the development of function after spinal cord transection in newborn rats. J. Neurosci. 17, 4856–4872. 9169544 10.1523/JNEUROSCI.17-12-04856.1997 PMC6573335

[B30] Mothe A. J. Tator C. H. (2013). Review of transplantation of neural stem/progenitor cells for spinal cord injury. Int. J. Dev. Neurosci. 31, 701–713. 10.1016/j.ijdevneu.2013.07.004 23928260

[B31] Pulizzi R. Musumeci G. Van den Haute C. Van De Vijver S. Baekelandt V. Giugliano M. (2016). Brief wide-field photostimuli evoke and modulate oscillatory reverberating activity in cortical networks. Sci. Rep. 6:e24701. 10.1038/srep24701 27099182 PMC4838830

[B32] Reier P. J. Bregman B. S. Wujek J. R. (1986). Intraspinal transplantation of embryonic spinal-cord tissue in neonatal and adult-rats. J. Comp. Neurol. 247, 275–296. 10.1002/cne.902470302 3522658

[B33] Reier P. J. Perlow M. J. Guth L. (1983). Development of embryonic spinal cord transplants in the rat. Brain Res. 312, 201–219. 10.1016/0165-3806(83)90137-2 6652515

[B34] Sharp K. G. Yee K. M. Steward O. (2014). A re-assessment of long distance growth and connectivity of neural stem cells after severe spinal cord injury. Exp. Neurol. 257, 186–204. 10.1016/j.expneurol.2014.04.008 24747827 PMC4123968

[B35] Silva N. A. Sousa N. Reis R. L. Salgado A. J. (2014). From basics to clinical: a comprehensive review on spinal cord injury. Prog. Neurobiol. 114, 25–57. 10.1016/j.pneurobio.2013.11.002 24269804

[B36] Stokes B. T. Reier P. J. (1992). Fetal grafts alter chronic behavioral outcome after contusion damage to the adult-rat spinal-cord. Exp. Neurol. 116, 1–12. 10.1016/0014-4886(92)90171-l 1559561

[B37] Tscherter A. Heuschkel M. O. Renaud P. Streit J. (2001). Spatiotemporal characterization of rhythmic activity in rat spinal cord slice cultures. Eur. J. Neurosci. 14, 179–190. 10.1046/j.0953-816x.2001.01635.x 11553271

[B38] von Bohlen Und Halbach O. (2007). Immunohistological markers for staging neurogenesis in adult hippocampus. Cell Tissue Res. 329, 409–420. 10.1007/s00441-007-0432-4 17541643

[B39] Watanabe K. Nakamura M. Iwanami A. Fujita Y. Kanemura Y. Toyama Y.. (2004). Comparison between fetal spinal-cord- and forebrain-derived neural stem/progenitor cells as a source of transplantation for spinal cord injury. Dev. Neurosci. 26, 275–287. 10.1159/000082144 15711067

[B40] Zhang F. Aravanis A. M. Adamantidis A. de Lecea L. Deisseroth K. (2007). Circuit-breakers: optical technologies for probing neural signals and systems. Nat. Rev. Neurosci. 8, 577–581. 10.1038/nrn2222 17643087

